# Dual Effects of Chinese Herbal Medicines on Angiogenesis in Cancer and Ischemic Stroke Treatments: Role of HIF-1 Network

**DOI:** 10.3389/fphar.2019.00696

**Published:** 2019-06-26

**Authors:** Ming Hong, Honglian Shi, Ning Wang, Hor-Yue Tan, Qi Wang, Yibin Feng

**Affiliations:** ^1^Institute of Clinical Pharmacology, Guangzhou University of Chinese Medicine, Guangzhou, China; ^2^Department of Pharmacology and Toxicology, University of Kansas, Lawrence, KS, United States; ^3^School of Chinese Medicine, Li Ka Shing Faculty of Medicine, The University of Hong Kong, Hong Kong

**Keywords:** angiogenesis, herbal medicine, hypoxia-inducible factor-1, cancer, ischemic stroke

## Abstract

Hypoxia-inducible factor-1 (HIF-1)–induced angiogenesis has been involved in numerous pathological conditions, and it may be harmful or beneficial depending on the types of diseases. Exploration on angiogenesis has sparked hopes in providing novel therapeutic approaches on multiple diseases with high mortality rates, such as cancer and ischemic stroke. The HIF-1 pathway is considered to be a major regulator of angiogenesis. HIF-1 seems to be involved in the vascular formation process by synergistic correlations with other proangiogenic factors in cancer and cerebrovascular disease. The regulation of HIF-1–dependent angiogenesis is related to the modulation of HIF-1 bioactivity by regulating HIF-1α transcription or protein translation, HIF-1α DNA binding, HIF-1α and HIF-1α dimerization, and HIF-1 degradation. Traditional Chinese herbal medicines have a long history of clinical use in both cancer and stroke treatments in Asia. Growing evidence has demonstrated potential proangiogenic benefits of Chinese herbal medicines in ischemic stroke, whereas tumor angiogenesis could be inhibited by the active components in Chinese herbal medicines. The objective of this review is to provide comprehensive insight on the effects of Chinese herbal medicines on angiogenesis by regulating HIF-1 pathways in both cancer and ischemic stroke.

## Introduction

Angiogenesis is the formation and remodeling of new blood vessels and capillaries from the existing vasculature through interaction among cellular matrix, cytokines, and proteases. It plays a pivotal role in diffusion exchange of metabolites and nutrients in all the tissues and organs of the human body (Shi, 2009; Kusumbe et al., 2014), occurring throughout our lives in both diseased and healthy states. Changes in metabolism result in proportional changes in angiogenesis and, therefore, proportional changes in capillarity. Oxygen is crucial for this process. Hypoxia occurs when there is reduced oxygen supply and/or increased oxygen demand. It is the principal physiological stimulus for inducing angiogenesis, which provides a stimulus-response pathway that tries to maintain adequate oxygenation in pathological status, such as tumor growth and ischemic stroke (Mengozzi et al., 2012; Brown, 2016). There has been great interest during the past decades in regulating angiogenesis as a therapeutic target for cancer and ischemic stroke. The current clinical application based on the principle of angiogenesis includes antiangiogenic therapy and proangiogenic therapy. Antiangiogenic therapy has been used for cancer treatment, which inhibits the delivery of oxygen and nutrients to cancer cells. On the other hand, proangiogenic therapies in ischemic stroke could be beneficial by increasing blood flow. Hypoxia-inducible factor-1 (HIF-1), a regulator of essential adaptive responses to hypoxia-induced angiogenesis, is highly expressed under hypoxic conditions, such as aggressive tumors and ischemic brains (Sendoel et al., 2010; Berlow et al., 2017). HIF-1 has been suggested to be an important target in treating cancer and ischemic stroke by regulating the transcriptional activity of its downstream genes. The activity and accumulation of HIF-1α protein were found to be regulated at different levels, such as regulating HIF-1α synthesis stability or transactivation throughout its life cycle inside the cells (Yeom et al., 2011; Soleymani Abyaneh et al., 2017).

Traditional Chinese herbal medicine has a long history of clinical use in both cancer and stroke treatments in Asia. Chinese herbal medicines often use a variety of herbs in different complex combinations to enhance their therapeutic effects or reduce their toxicity. Growing evidence has demonstrated potential proangiogenic benefits of Chinese herbal medicines in ischemic stroke whereas tumor angiogenesis could be inhibited by the active components in herbal medicines (Hong et al., 2015; Gandin et al., 2016; Hong et al., 2016; Guo et al., 2018). Thus, the objective of this review is to provide comprehensive insight on how Chinese herbal medicines impact angiogenesis by regulating HIF-1 pathways in both cancer and ischemic stroke. In this study, we tried to give a systematic and timely update about the effects and mechanisms of several Chinese herbal medicines targeting HIF-1 pathways in cancer or ischemic stroke, such as Xue-Fu-Zhu-Yu decoction, ginsenosides, Pien Tze Huang, Yi Ai Fang, baicalein, and curcumin. Their mechanisms of antiangiogenesis or proangiogenesis behaviors, potential toxicity, or side effects and future research directions were discussed.

## Method

Both clinical trials and basic research on Chinese herbal medicines that target the HIF-1 pathway were included to assess their efficacy and underlying mechanisms. One Chinese database (China Journals Full-Text Database) and four English databases (AMED, MEDLINE, EMBASE, and The CENTRAL) were applied in our study to retrieve more recent publications on this topic. Chinese herbal medicines and their active compounds for ischemic stroke or cancer treatment will be included in this review paper if more than two research papers have described the *in vitro* and *in vivo* studies of the particular subject or of any paper describing clinical trials on the subject.

## Hypoxia-Induced Angiogenesis

**Figure 1:**
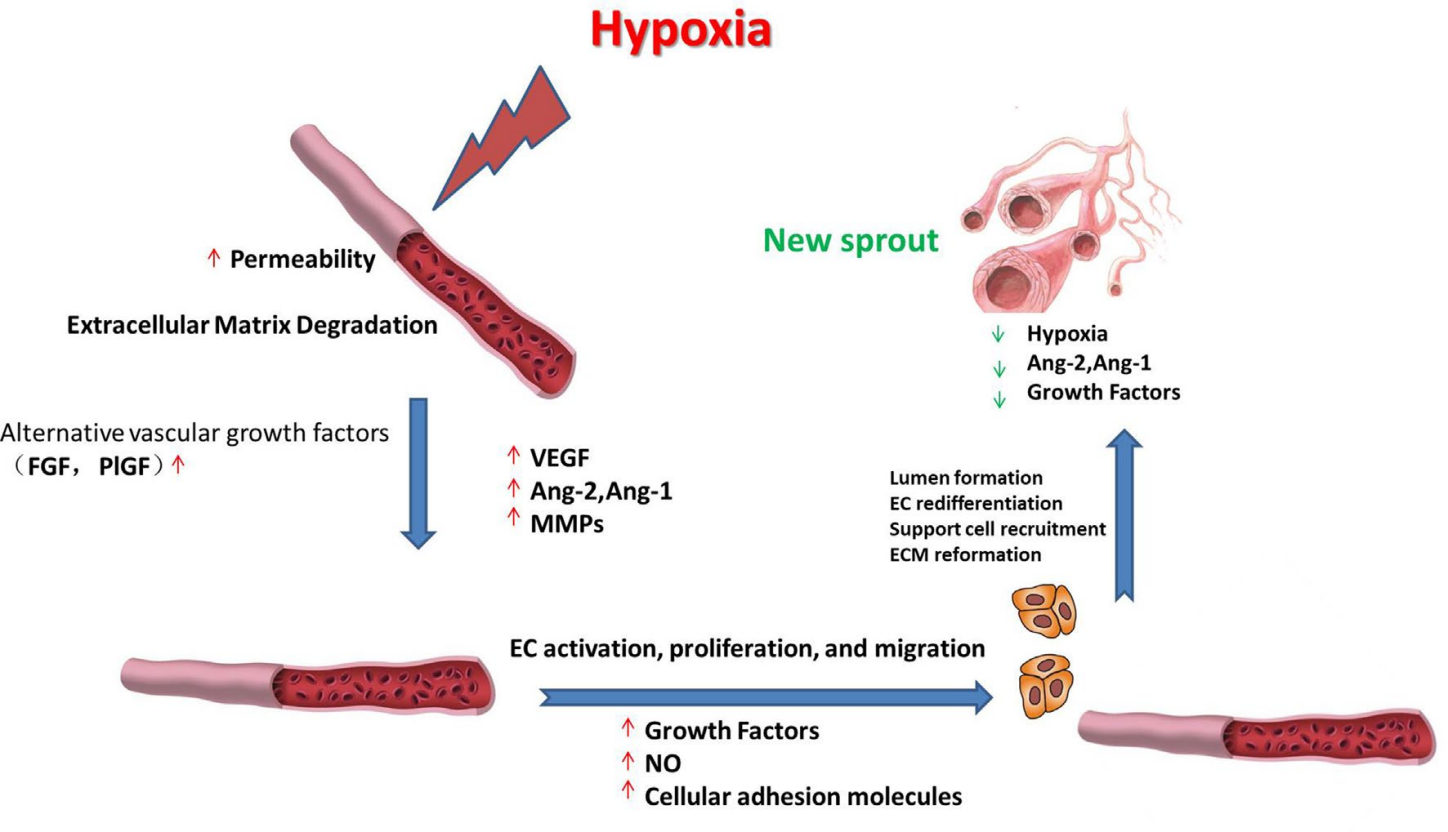
Schematic representation of the roles of vascular endothelial growth factor (VEGF), angiopoietin-1 (Ang-1), angiopoietin-2 (Ang-2), matrix metalloproteinases (MMPs), and various growth factors during hypoxia-induced angiogenesis. The processes include upregulating the expression of proangiogenic factors; the synergistic effects of VEGF, Ang-1, and Ang-2 on angiogenesis; degrading the matrix components; new vessel formation; and stabilization, as described in detail in the text.

Hypoxia is the nonphysiological exposure to low oxygen tension of cells or tissues, which is associated with various pathological events, such as stroke, inflammation, and cancer. These pathological events induce the restoration of oxygen homeostasis by activating repair mechanisms such as angiogenesis. Hypoxia-induced angiogenesis includes several steps (**Figure 1**). 1) Exposure to low oxygen tension upregulates the expression of proangiogenic growth factors that activate their receptors (Sendoel et al., 2010; Berlow et al., 2017). 2) Vascular permeability increases in response to vascular endothelial growth factor (VEGF), thereby inducing the exudation of plasma proteins that form a primitive scaffold for migrating endothelial cells. Angiopoietin-1 (Ang-1) and angiopoietin-2 (Ang-2) exhibit antagonistic properties during the development of the vessel. Ang-1 is critical for vessel maturation, adhesion, migration, and survival, whereas Ang-2 is involved in vessel destabilization and promoting cell death. Yet, when it is in conjunction with VEGFs, Ang-2 can promote neovascularization (Jain and Carmeliet, 2012). The matrix metalloproteinases (MMPs) such as MMP2 and MMP9 can further induce angiogenesis by degrading matrix components (Ota et al., 2009; Kang et al., 2012). 3) Proliferative endothelial cells assemble and form a lumen by migrating to a distant location (Nieuwenhuis et al., 2017). In this stage, several proteins can promote endothelial cell survival, adhesion, and migration, such as VE-cadherin and integrins αβ. After new vessels are formed, pericytes and smooth muscle cells will stabilize the walls and prevent leakage by surrounding the novel capillaries. Other factors including Ang-1 and platelet-derived growth factor receptor (PDGFR) also take part in the maturation of novel capillaries (Rivera and Bergers, 2014).

Hypoxia-induced angiogenesis shows significant differences in signal pathways compared with physiological angiogenesis. For example, physiological angiogenesis in embryonic development requires activating the VEGF pathway, whereas hypoxia-induced angiogenesis such as tumor angiogenesis can also induce angiogenesis by recruiting myeloid cells and upregulate alternative vascular growth factors in addition to VEGF, such as fibroblast growth factor (FGF) and placental growth factor (PlGF). Although postischemic tissue revascularization is crucial for recovery in brain tissues after ischemic stroke (Li Q. et al., 2018) or in the heart after myocardial infarction (Chen R. et al., 2018), the activation of angiogenesis is harmful in disorders such as macular degeneration and cancer (Pio et al., 2013). Therefore, there is great interest in regulating angiogenesis as a possible therapeutic method for different kinds of diseases. Elucidating the molecular mechanism of hypoxia-induced angiogenesis will help in the identification of potential therapeutic targets and improve therapeutic effects.

## Hypoxia-Inducible Factor-1

**Figure 2:**
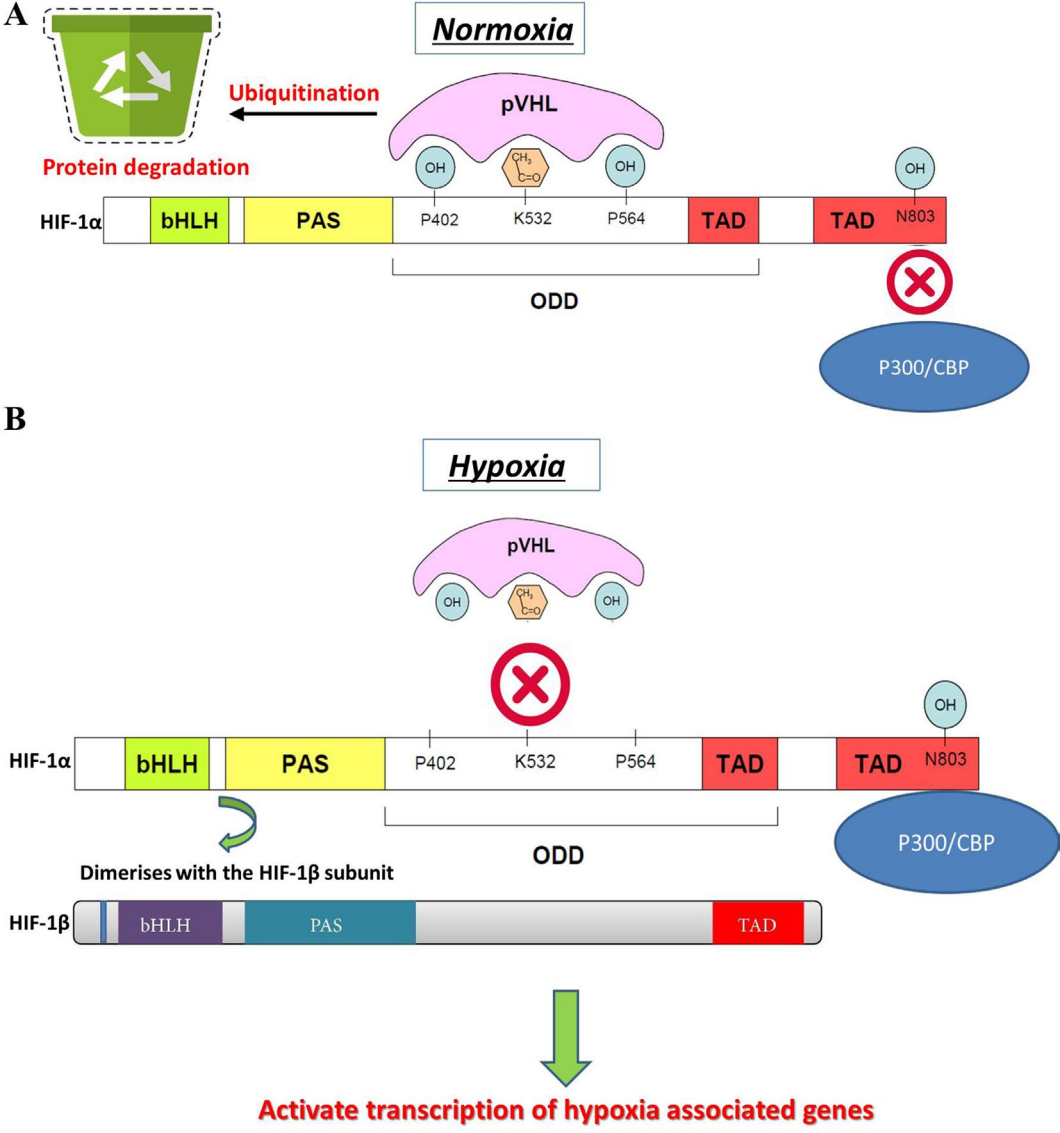
HIF-1α gene structure, stability, and activation. **(A)** Normal oxygen level induces the degradation of HIF-1α by hydroxylation or acetylation-mediated VHL binding and also transcriptional activity of HIF-1α. **(B)** Under hypoxic conditions, VHL is not prolyl-hydroxylated and cannot bind to HIF-1α protein, which leads to a decreased rate of HIF-1α degradation. Hypoxia promotes the interaction of HIF-1α within CBP/p300 and induces dimerization of HIF-1α with HIF-1β, which results in HIF-1 transcription factor formation. The active HIF-1 will further bind to HREs and activate the transcription of downstream genes.

Changes in oxygen supply represent a pivotal physiological stimulus for all eukaryotic cells that require adequate oxygen consumption for intracellular metabolic reactions. In addition to its contribution to the maintenance of intracellular bioenergetics by producing mitochondrial ATP, O_2_ also serves as a universal electron acceptor in various biochemical pathways. Therefore, genes involved in responding to hypoxia are highly conserved during evolution. HIF-1 is an oxygen-dependent transcriptional activator, which is composed of HIF-1α, the alpha subunit, and the aryl hydrocarbon receptor nuclear translocator (Arnt), the beta subunit. Both subunits belong to the bHLH-PAS (Per/Arnt/Sim) family. HIF-1 is induced in hypoxic cells and binds to the cis-acting hypoxia response element (HRE) of the human EPO gene, which is required for erythropoietin synthesis (Aldo and Elisabetta, 2018; Zhu and Zhang, 2018). Intracellular oxygen concentration levels can affect the subcellular localization and protein activity of the HIF-1α subunit, whereas the expression of HIF-1β is not regulated by the oxygen level (Wang et al., 2018). The HIF-1α and HIF-1β subunits are similar in structure, and both contain two PAS domains. The bHLH and PAS domains are critical for the heterodimer formation of HIF-1α and HIF-1β and for DNA binding. The HIF-1α subunit contains N-terminal transactivation domains (TAD-N) and C-terminal transactivation domains (TAD-C) concatenated by an inhibitory domain (**Figure 2**). The TAD-N is continuous with protein stability that overlaps with the oxygen-dependent degradation (ODD) domain. The TAD-C is independent of protein stability that interacts with p300/CBP and is critical for transcription activity. The HIF-1α protein is unstable (half-life = 5 min) and is modified by various posttranscriptional regulations, including phosphorylation, hydroxylation, ubiquitination, acetylation, and nitrosation. Factor inhibiting HIF-1 (FIH-1) hydroxylates asparagine-803 of HIF-1α within the TAD-C under normoxic conditions, which inhibits the interaction of HIF-1α with transcriptional coactivators. The molecular mechanisms of the pivotal role of HIF-1 in the regulation of angiogenesis have been revealed in recent years. Recent studies have demonstrated that HIF-1 activity in human tissues can induce angiogenesis in the following ways: 1) by activating the transcription of various angiogenic genes or their receptors such as ANGPT1, ANGPT2, VEGF, PlGF, and PDGFB (Chen et al., 2017); 2) by modulating proangiogenic chemokines and receptors (SDF-1α, sphingosine-1-phosphate, stromal cell–derived factor 1α, receptor CXCR4, sphingosine-1-phosphate receptors, and C-X-C chemokine receptor type 4), thus promoting the recruitment of endothelial progenitor cells to the hypoxic site (Soni and Padwad, 2017); and 3) by facilitating cell cycle progression and DNA replication in endothelial cells (Toth and Warfel, 2017). Through the phosphoinositide 3-kinase (PI3K) or Ras/MAPK pathway, several growth factors and their cognate receptors can influence cellular responses to hypoxia and regulate the expression of HIF-1α. Previous studies have shown that inhibition of PI3K pathway downregulates both basal and mitogen-induced HIF-1α expression (Cheng et al., 2018). In general, the modifications of HIF-1 are rapidly and precisely regulated according to the cellular oxygen concentration by multiple signaling. The hypoxia-induced angiogenesis is a highly complex and orchestrated process in human disease. HIF-1 was found to be a major modulator of hypoxia-induced angiogenesis by synergistic correlations with various proangiogenic factors and regulates many genes that play important roles in angiogenesis (**Table 1**). Thus, HIF-1 modulation could offer therapeutic benefits for various hypoxia pathologies, including diseases with high mortality and morbidity rates, such as cancer and ischemic stroke.

**Table 1:** Angiogenesis-Related Genes That Are Transcriptionally Activated by HIF-1.

Target Gene	Protein Name	Roles in Angiogenesis	Reference
c-MET	c-mesenchymal-epithelial transition	Promotes endotheliocyte motility and vascular formation	(Matteucci et al., 2003)
LRP1	Low-density lipoprotein receptor-related protein 1	Regulates vascular integrity	(Woodley et al., 2009; Li et al., 2014)
HO-1	Heme oxygenase-1	Regulates vascular tone and blood pressure	(Sato et al., 2012; Mathew and Sarada, 2018)
GPI	Glucose-6-phosphate isomerase	Tumor-secreted cytokine that stimulates vascular endothelial cell motility	(Zdralevic et al., 2018)
MIC2	CD99 antigen	Inhibits cell-extracellular matrix adhesion and promotes vascular remodeling	(Ohradanova et al., 2008; Llurba et al., 2014)
VEGF	Vascular endothelial growth factor	Stimulates the formation of blood vessels	(Al-Anazi et al., 2018; Fortenbery et al., 2018; Prangsaengtong et al., 2018)
EG-VEGF	Endocrine gland–derived vascular endothelial growth factor	Angiogenic growth factor specifically expressed in the ovaries	(Su et al., 2014; Mi et al., 2018)
ENG	Endoglin	Regulates transforming growth factor-β–dependent vascular remodeling and angiogenesis	(Bluff et al., 2009; Tal et al., 2010)
ET1	Endothelin 1	Regulates vascular tone and blood pressure	(Kaul et al., 2013; Ambrosini et al., 2015; Belaidi et al., 2016)
LEP	Leptin	Has mitogenic activity on vascular endothelial cells and plays a role in matrix remodeling by regulating the expression of matrix metalloproteinases (MMPs) and tissue inhibitors of metalloproteinases (TIMPs)	(Al-Anazi et al., 2018; Rausch et al., 2018)
TGF-β3	Transforming growth factor beta 3	Regulates angiogenesis in the developing brain *via* paracrine signaling to vascular epithelial cells	(Taheem et al., 2018; Tsai et al., 2018)
α_1β_-AR	α_1β_-adrenergic receptor	Activates vascular epithelial cell proliferation	(Park et al., 2011; Forbes et al., 2016)
ADM	Adrenomedullin	Regulates vascular tone and blood pressure	(Sena et al., 2014; Matsumoto et al., 2018)
NOS2	Nitric oxide synthase 2	Regulates vascular tone and blood pressure	(Magierowski et al., 2018; Pena-Mercado et al., 2018; Suvanish Kumar et al., 2018)
TFF	Intestinal trefoil factor	Regulates vascular epithelial restitution	(Miki et al., 2004; Manresa and Taylor, 2017)
MMP2	Matrix metallopeptidase 2	Regulates vascular patterning and branching	(Sharma et al., 2018; Tyszka-Czochara et al., 2018)
PDGFβ	platelet-derived growth factor receptor-β	Maintains vascular stability	(Beppu et al., 2005; Gramley et al., 2010; Hsu et al., 2014)
FN1	Fibronectin 1	Promotes vascular remodeling	(Kondisetty et al., 2018; Zeinali et al., 2018)
PAI-1	plasminogen activator inhibitor-1	Promotes vascular remodeling	(Kabei et al., 2018; Peterle et al., 2018; Toullec et al., 2018)
UPAR	Urokinase-type plasminogen activator receptor	Regulates growth factor activation; promotes ECM and vascular remodeling	(Carroll and Ashcroft, 2006; Laurenzana et al., 2017)
P4H (I)	prolyl-4-hydroxylase (i)	Regulates vascular collagen production	(Trollmann et al., 2018)
ANGPT2	Angiopoietin-Tie2	Regulates vascular remodeling	(Yamakawa et al., 2004; Trollmann et al., 2018)
KRT19	keratin-19	responsible for the structural integrity of vascular ECs	(Copple, 2010)
KRT14	keratin-14	Responsible for the structural integrity of vascular ECs	(Pahlman et al., 2015)
KRT18	keratin-18	Responsible for the structural integrity of vascular ECs	(Muller et al., 2018)

## Hypoxia-Induced Angiogenesis in Cancer and the Role of Hif-1

Because of the expansive growth activities within malignant tumor, cancer cells are highly metabolic. However, the poorly vascularized original tissue structure leads to inadequate oxygen supply for tumor progression. Hypoxia is commonly observed in the microenvironment of cancer, which arises in cancer *via* the uncontrolled proliferation driven by the oncogene of cancer cells in the absence of an efficient vascular bed. As a result of rapid cell proliferation, the cancer cell quickly exhausts the oxygen supply and nutrient from the normal vasculature, which leads to hypoxia. In previous studies, the relationship between hypoxia and tumor progression has been proven by O_2_-sensitive microsensors (Semenza, 2003; Bohonowych et al., 2011). Clinical studies have shown that patients with hypoxic cervical tumors, head and neck cancer, and sarcoma of soft tissue may have worse disease-free survival than that of patients with normally aerated tumors. The inadequate oxygen supply at the tumor tissue may induce tumor progression through selective pressure by the mutation of cancer suppressor genes, which may reduce tumor cells’ apoptotic capacity and promote tumor growth. Another key characteristic of the hypoxic response in tumor is the modulation of multiple genes that promote angiogenesis to fortify oxygen supply (Zagzag et al., 2000).

Cancer growth and metastasis depend on lymphangiogenesis and neovascularization triggered by hypoxia signals from cancer cells. Cancer cells under hypoxic conditions will upregulate the expression of PDGF, Ang-2, stromal-derived factor 1 (SDF-1), and VEGF, which are crucial in endothelial cell activation and promoting neoangiogenesis. Activated HIF-1 plays a crucial role in hypoxia-adaptive responses of the tumor cells through transcriptional activation of these proangiogenesis genes. As shown in previous studies, HIF-1 can mediate acute hypoxia-induced VEGF expression in neuroblastoma, whereas HIF-2 modulates VEGF expression during prolonged hypoxia (Maxwell et al., 1999). Furthermore, VEGF expression under hypoxia may increase the activity of other proangiogenic factors and their receptors; thus, vessel outgrowth was stimulated through multiple factors. This so-called “angiogenic switching” induces tumor angiogenesis and stimulates tumor growth by supplying nutrients and oxygen by newly formed vessels (Singh et al., 2017). During the cellular adaptation to hypoxic stress, PI3K/AKT/mTOR and MAPK signaling pathways are involved in hypoxia-induced tumor angiogenesis by various growth factors that bind to toll-like receptors (TLRs), alarmin receptors, receptor tyrosine kinases, and G protein–coupled receptors on cell surface, which may also activate HIF-1 (De Francesco et al., 2018). The mitogen-activated protein kinase (MAPK) and PI3K pathways are activated by the combination of growth factor with its cognate receptor tyrosine kinase. PI3K promotes the activation of the downstream mammalian target of rapamycin (mTOR) and serine/threonine kinase AKT. mTOR further induces p70 S6 kinase (S6K) and its substrate phosphorylation then induces HIF-1α protein synthesis. In the MAPK pathway, the extracellular signal-regulated kinase (ERK) is activated by the upstream signal cascade (RAS/RAF/MEK). Activated ERK promotes the phosphorylation of eukaryotic translation initiation factor 4E (eIF-4E) binding protein (4E-BP1) and MAP kinase interacting kinase (MNK). MNK can also phosphorylate eukaryotic translation initiation factor 4E (eIF-4E) directly. Then, the HIF-1α mRNA translation is activated (Rius et al., 2008; Ban et al., 2017; Aldo and Elisabetta, 2018). Key cellular responses to the hypoxic tumor microenvironment triggered by HIF-1 and its downstream targets increase the vascular formation, cancer invasiveness, and resistance to treatment (Liu H. et al., 2018).

Hypoxia-induced tumor angiogenesis is stimulated and regulated by both activator and inhibitor molecules. However, simple upregulation of the activity of proangiogenesis factors is not sufficient for neovascularization of the tumor. Negative regulators or endogenous inhibitors of vessel growth also need to be downregulated, such as the thrombospondin-1 and thrombospondin-2. In recent years, various anticancer agents have been developed by targeting these angiogenic activator or inhibitor molecules in malignant tumor. A number of antiangiogenesis drugs have been approved by the U.S. Food and Drug Administration (FDA) for treating progressive cancer. So far, most of these drugs are molecular targeted agents that were developed specifically to target VEGF or its receptors, such as bevacizumab (Avastin) and vandetanib (Caprelsa) (Li et al., 2018a). During the last two decades, interest in the role of HIF-1 in tumor angiogenesis has grown exponentially since its identification and molecular characterization in human cancer. Much progress has been made recently about the cellular and molecular mechanism of HIF-1 and its involvement in cancer growth and metastasis based on the analysis of experimental animal models and human cancer biopsies.

In brief, activation of HIF-1 in cancer cells is one of the key masters orchestrating their adaptation mechanism to the hypoxic conditions. Considering the pivotal roles of HIF-1 in tumor angiogenesis, there has been great interest in developing novel anticancer agents inhibiting the related pathway. As we know, HIF-1 modulation in cancer cells is a complex network including various signal cascades and overlapping mechanisms, each of which might act as a potential target to selectively intervene cancer.

## Chinese Herbal Medicines Mediate Antiangiogenic Factors by Regulating Hif-1 Pathways in Cancer Treatment

**Figure 3:**
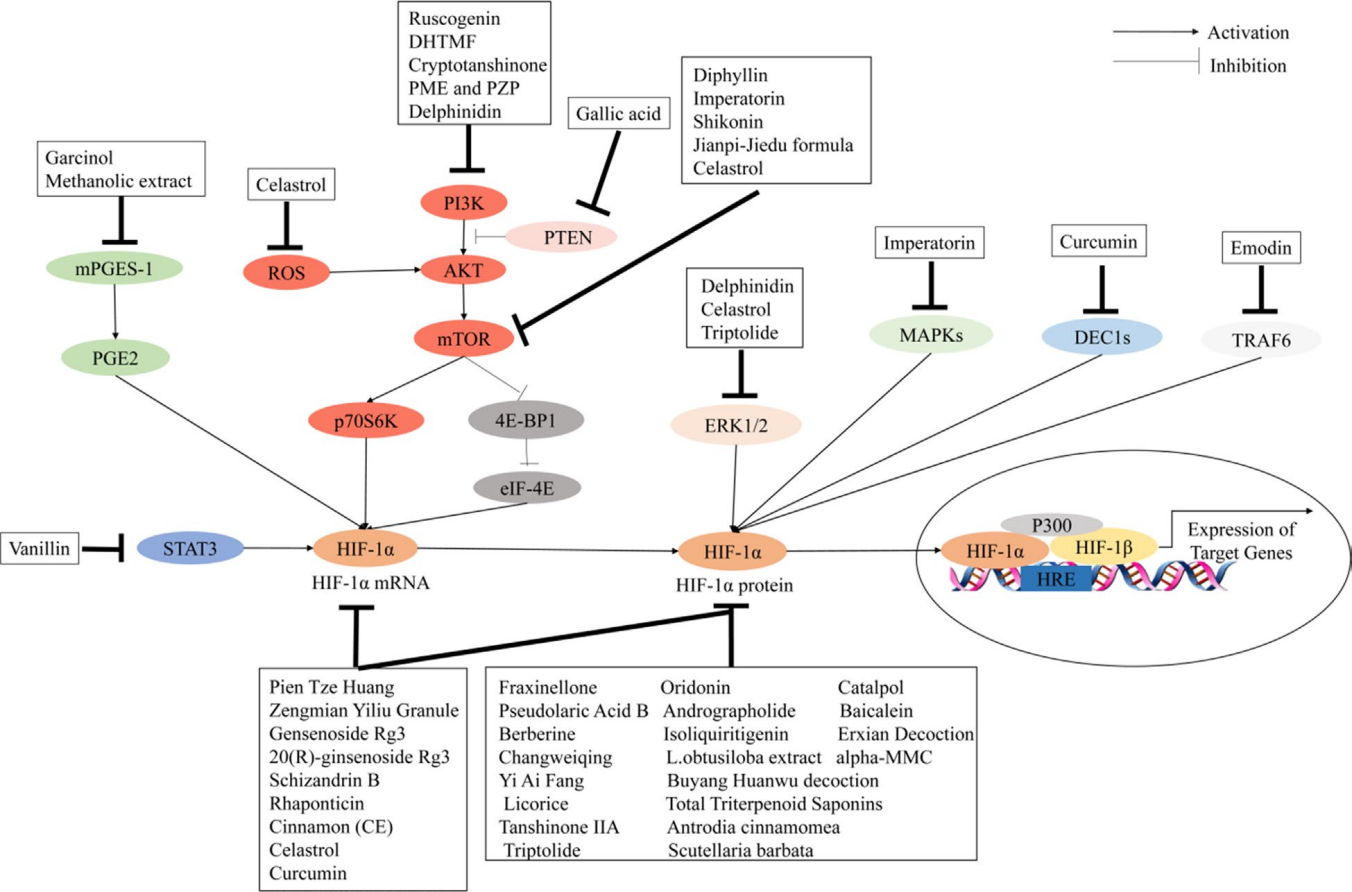
Chinese herbal medicines inhibit the activation of the HIF-1 pathway in cancer treatment through different targets.

The use of Chinese herbal medicines to treat cancer dates back centuries in ancient traditional folklore in China and Asian countries (Qin et al., 2018; Oyenihi and Smith, 2019). Many herbal extracts and herbal soups have been reported that could relieve clinical symptoms, improve quality of life, and reduce side effects in cancer therapy (Dong et al., 2010; Xu et al., 2014; Tian et al., 2010). In view of the importance of HIF-1 in tumor angiogenesis, the development of herbal medicine inhibitors for this pathway has attracted wide interest. It is clear that the regulation of HIF-1 is a highly complex network cascade and overlapping mechanisms involving multiple targets and signaling pathways, such as HIF-1α mRNA expression, HIF-1α protein expression, and HIF-1 transcriptional activity. As shown in **Figures 2** and **3**, we have concluded that Chinese herbal medicines can regulate HIF-1 by targeting different targets that exert antiangiogenic effects in cancer therapy.

### Inhibitors of HIF-1α mRNA and/or Protein Expression

**Figure 4:**
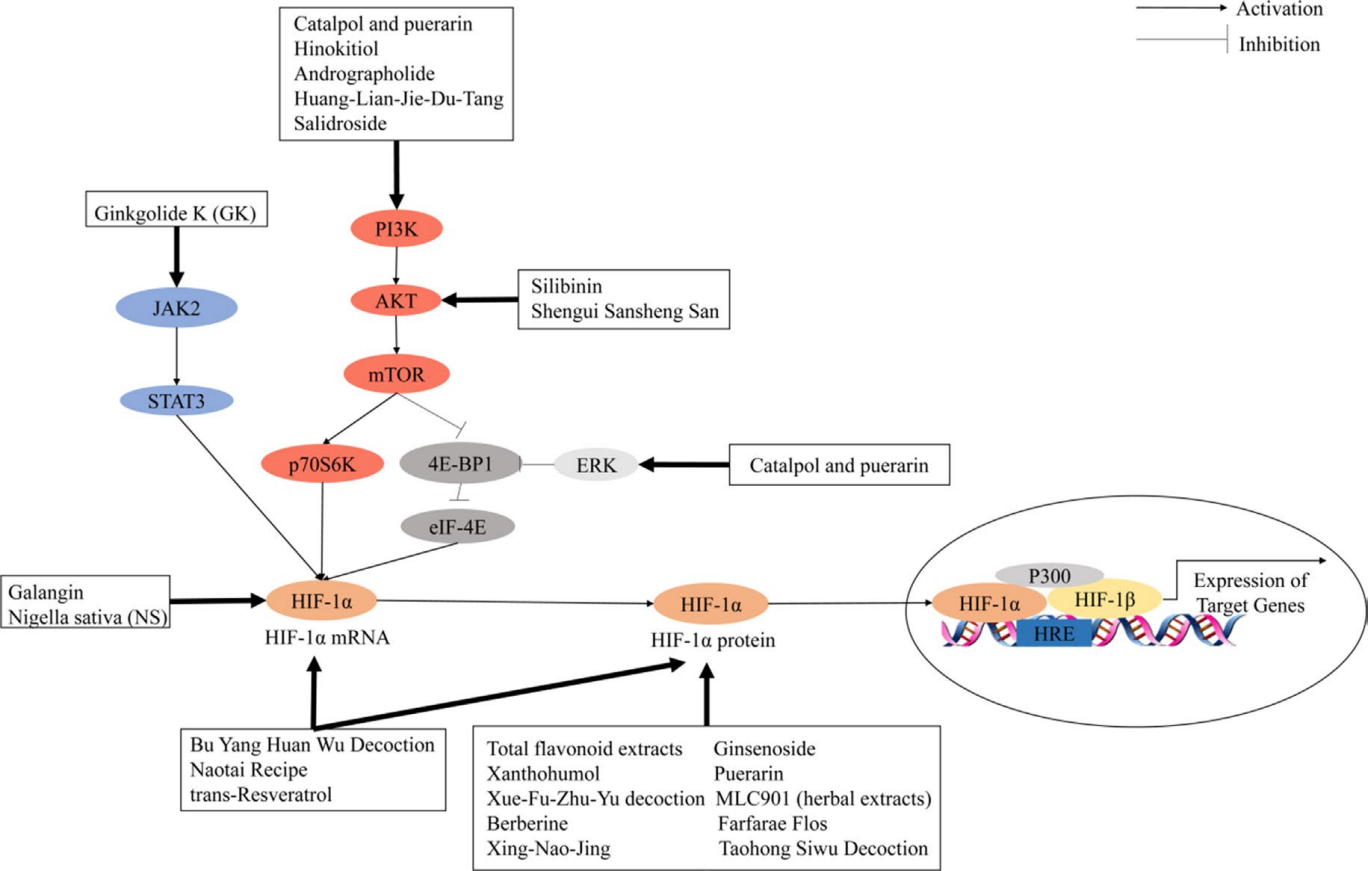
Herbal medicines promote the activation of the HIF-1 pathway through different targets in ischemic stroke.

Numerous herbal medicines that inhibit HIF-1α mRNA and/or protein expression have significant antiangiogenic effects. Berberine, the main active ingredient isolated from *Coptis chinensis*, has been shown to decrease the expression of HIF-1α and VEGF in esophageal cancer, hepatocellular carcinoma, prostate cancer, nasopharyngeal carcinoma, and lung cancer (Fu et al., 2013; Yang et al., 2013; Tsang et al., 2015; Zhang et al., 2014a; Zhang C. et al., 2014). Isoliquiritigenin, a natural product derived from liquorice, could significantly decrease VEGF expression by promoting HIF-1α degradation in breast cancer cells (Wang et al., 2013). Ginsenoside Rg3 is one of the active ingredients in ginseng. Chen et al. (2010) reported that ginsenoside Rg3 could inhibit VEGF expression through downregulation of HIF-1α protein in various human cancers. Wang et al. (2009) reported that ginsenoside Rg3 could inhibit HIF-1α and VEGF expression during hypoxia and inhibit hep-2 cell growth by affecting cell cycle progression. Another report has shown that 20(R)-ginsenoside Rg3 could inhibit tumor angiogenesis by suppressing the expression of VEGF, MMP9, and HIF-1α in a mouse model of Lewis lung cancer (Geng et al., 2016). Schisandrin B (Sch B) is the most abundant dibenzocyclooctadiene lignan in *Schisandra chinensis*. Lv et al. (2015) found that Sch B could inhibit the migration and invasion of A549 cells by decreasing the expressions of HIF-1, VEGF, MMP-2, and MMP-9 *in vitro*. *Scutellaria barbata* is widely used in the treatment of cancer in traditional Chinese medicine. Shiau et al. (2014) found that *S. barbata* could play an antiangiogenic role by targeting the HIF-1α signaling pathway and reducing the expression of VEGF. Hu et al. (2012) used a mouse model of ovarian carcinoma xenograft to study the underlying anticancer mechanisms of Zengmian Yiliu granule (ZMYLG), a traditional Chinese formula. ZMYLG could downregulate the protein expression and mRNA of HIF-1α and VEGF and exert antiangiogenic effects on ovarian carcinoma xenografts. Triptolide (TPL) is an active ingredient extracted from triptolide and widely used in cancer treatment. Li et al. (2018b) found that TPL could inhibit angiogenesis by reducing the expression of HIF-1α and VEGF in a dose-dependent manner. Protein alpha-momorcharin (alpha-MMC) is isolated from seeds of the bitter gourd *Momordica charantia*. Pan et al. (2014) showed that alpha-MMC has significant inhibitory effects on normal and hypoxic nasal-pharyngeal cancer cells by blocking HIF-1α signaling such as the expression of VEGF and UPR. Baicalein, a type of flavonoid isolated from the roots of *Scutellaria baicalensis*, could suppress tumor growth, which is associated with a reduction of HIF-1α and VEGF in an orthotopic glioma mouse model (Wang and Jiang, 2015) (**Figure 4**). **Table 2** lists important Chinese herbal medicines that act on HIF-1 mRNA and/or protein expression.

**Table 2:** Chinese Herbal Medicines and Their Molecular Targets That Are Regulated by the HIF-1 Pathway in Cancer.

Herb Medicine	Molecular Target	Reference
Inhibitors of HIF-1 mRNA and/or protein expression		
Scutellaria barbata	HIF-1α protein expression	(Shiau et al., 2014)
Antrodia cinnamomea	HIF-1α protein expression	(Liu et al., 2013)
*L. obtusiloba* extract	HIF-1α protein expression	(Freise et al., 2011)
Pien Tze Huang	HIF-1α mRNA and protein expression	(Chen et al., 2015)
Yi Ai Fang	HIF-1α protein expression	(Hou et al., 2016)
Changweiqing	HIF-1α protein expression	(Li et al., 2011)
Erxian decoction	HIF-1α protein expression	(Yu et al., 2012)
Zengmian Yiliu granule	HIF-1α mRNA and protein expression	(Hu et al., 2012)
Buyang Huanwu decoction	HIF-1α protein expression	(Min et al., 2016)
Fraxinellone	HIF-1α protein expression	(Xing et al., 2018)
Oridonin	HIF-1α protein expression	(Li C. et al., 2018)
Catalpol	HIF-1α protein expression	(Zhu et al., 2017)
Pseudolaric acid B	HIF-1α protein expression	(Wang et al., 2017)
Andrographolide	HIF-1α protein expression	(Shi et al., 2017)
Baicalein	HIF-1α protein expression	(Wang and Jiang, 2015)
Berberine	HIF-1α protein expression	(Fu et al., 2013; Yang et al., 2013; Zhang et al., 2014a; Zhang C. et al., 2014; Tsang et al., 2015)
Isoliquiritigenin	HIF-1α protein expression	(Wang et al., 2013)
alpha-MMC	HIF-1α protein expression	(Pan et al., 2014)
Total triterpenoid saponins	HIF-1α protein expression	(Jia et al., 2017)
Licorice	HIF-1α protein expression	(Park et al., 2016)
Tanshinone IIA	HIF-1α protein expression	(Fu et al., 2014; Sui et al., 2017)
Triptolide	HIF-1α protein expression	(Li et al., 2018b)
Ginsenoside Rg3	HIF-1α mRNA and protein expression	(Wang et al., 2009; Chen et al., 2010)
20(R)-ginsenoside Rg3	HIF-1α mRNA and protein expression	(Geng et al., 2016)
Schizandrin B	HIF-1α mRNA and protein expression	(Lv et al., 2015)
Rhaponticin	HIF-1α mRNA and protein expression	(Kim and Ma, 2018)
Cinnamon (CE)	HIF-1α mRNA and protein expression	(Zhang et al., 2017)
Celastrol	HIF-1α mRNA and protein expression	(Huang et al., 2011)
Curcumin	HIF-1α mRNA and protein expression	(Bae et al., 2006; Das and Vinayak, 2014; Li et al., 2018a)
Inhibitors of HIF-1 transcriptional activity		
Triptolide	HIF-1α transcriptional activity	(Zhou et al., 2010)
Scutellaria barbata	HIF-1α transcriptional activity	(Shiau et al., 2014)
Inhibitors of signal transduction pathways		
Methanolic extract	mPGES-1–PGE2–HIF-1α	(Ranjbarnejad et al., 2017a)
Jianpi-Jiedu formula	mTOR–HIF-1α–VEGF	(Mao et al., 2016)
Vanillin	STAT3–HIF-1α	(Park et al., 2017)
Garcinol	mPGES-1–PGE2–HIF-1α	(Ranjbarnejad et al., 2017b)
Diphyllin	mTORC1–HIF-1α–VEGF	(Chen H. et al., 2018)
Imperatorin	mTOR–p70S6K–4E-BP1, MAPK	(Mi et al., 2017)
Shikonin	mTOR-p70S6K–4E-BP1	(Li M. et al., 2017)
Tanshinone IIA	mTOR-p70S6K-4E-BP1	(Wang X. et al., 2017)
Celastrol	mTOR-p70S6K–eIF4E, ERK1/2	(Ma et al., 2014)
5,3’-Dihydroxy-6,7,4’-trimethoxyflavanone (DHTMF)	PI3K-Akt-mTOR	(Kim et al., 2015)
Ruscogenin	PI3K-Akt-mTOR	(Hua et al., 2018)
Cryptotanshinone	PI3K-Akt-mTOR	(Zhang et al., 2018)
PME and PZP	PI3K-Akt	(Sathya et al., 2010)
Delphinidin	PI3K-Akt-mTOR-p70S6K, ERK	(Kim et al., 2017)
Gallic acid	PTEN–AKT–HIF-1α	(He et al., 2016)
Emodin	TRAF6–HIF-1α–VEGF	(Shi and Zhou, 2018)
Celastrol	ROS-Akt-p70S6K	(Han et al., 2014)
Triptolide	ERK1/2–HIF-1α	(Liu H. et al., 2018)
Curcumin	DEC1–HIF-1α	(Wang X. et al., 2017)

### Inhibitors of HIF-1 Transcriptional Activity

So far, several herbal medicines have been shown to inhibit tumor angiogenesis through downregulating HIF-1 activation by inhibiting its transcriptional activity. Shiau et al. explored the underlying mechanisms of *S. barbata* on regulating HIF-1–dependent expression of VEGF. Hypoxia induces angiogenesis by upregulating VEGF expression. However, after treatment with *S. barbata*, the expression of VEGF was downregulated in lung cancer cells. In addition, *S. barbata* inhibited the proliferation and migration of endothelial cells under a hypoxic environment. *S. barbata* suppressed the transcriptional activity of HIF-1α and promoted the phosphorylation of the upstream signal molecule AKT (Shiau et al., 2014). Triptolide is the major active compound in traditional Chinese medicine (TCM) herb *Tripterygium wilfordii* Hook F. Triptolide exhibits significant chemotherapeutic effects against cancer based on its antiangiogenesis and drug resistance circumvention activities. Various biological molecules suppressed by triptolide have been identified as its potential targets. Triptolide could downregulate the transcriptional activity of HIF-1α and further decrease the transcriptional activity of its target genes including VEGF (Zhou et al., 2010).

### Inhibitors of Signal Transduction Pathways

Several Chinese herbal medicines have been reported to act on different signaling pathways to indirectly regulate HIF-1 activation and exert antiangiogenic effects in cancer treatment. Imperatorin is an active natural furocoumarin ingredient from *Angelica dahurica*. Mi et al. (2017) reported that imperatorin administration could inhibit tumor growth and tumor angiogenesis *in vivo* and *in vitro* and downregulate HIF-1α activation by targeting the mTOR/p70S6K/4E-BP1 and MAPK pathways. 5,3’-Dihydroxy-6,7,4’-trimethoxyflavanone (DHTMF) is one of the main ingredients of *Vitex rotundifolia*. Kim et al. (2015) showed that DHTMF could inhibit angiogenesis and induce apoptosis by decreasing the expression levels of HIF-1α and VEGF *via* the Akt/mTOR pathway in cancer cells. Kim et al. (2017) reported that diphyllin, a natural component of traditional Chinese medicine, could regulate the mTORC1/HIF-1α/VEGF pathway in the treatment of esophageal cancer (Kim et al., 2017). Curcumin is an active molecule isolated from the dried rhizome of *Curcuma longa*. Wang D. et al. (2017) found that curcumin could downregulate the HIF-1α, VEGF, DEC1, and STAT3 signal transduction pathways in the treatment of gastric cancer. Garcinol (camboginol) is a natural polyisoprenylated benzophenone isolated from dried rind of the *Garcinia indica*. Ranjbarnejad et al. (2017a) found that garcinol could inhibit VEGF, MMP2/9, and CXCR4 expression by targeting the mPGES-1/PGE2/HIF-1a pathway. Herbal medicines targeting signal transduction pathways are reported in **Table 2**.

## Activation of Hif-1–Dependent Angiogenesis in Ischemic Stroke

Stroke is one of the major causes of death and long-term disability worldwide. About 50% of patients who have suffered from a stroke live less than 1 year (Zhang and Chopp, 2009). There are two main types of stroke: ischemic and hemorrhagic. Ischemic strokes account for about 85% of all strokes, which is caused by a sudden halt of blood supply to the brain tissue because of ischemia and can result in permanent brain injury (Senior, 2001). The thrombotic or embolic occlusion of a cerebral artery will lead to irreversible neuronal cell death and further induce serious brain injury at the core of the infarct immediately. In addition, the secondary injury will result in the expansion of the area of brain injury, which can continue for an extended period after the first ischemic attack (Chopp and Li, 2002). Thus, reestablishment of the functional cerebral microvasculature network will improve regional blood supply and promote stroke recovery. Angiogenesis is a fundamental pathological process in malignant tumor growth and development. However, it may also occur as an advantageous defense response against hypoxia in ischemic stroke by improving blood supply to the brain tissue. Previous research has shown that angiogenesis is positively correlated to the survival rate of ischemic stroke patients, indicating that regulation of the neovascular growth in the ischemic brain could be a pivotal target for ischemic stroke treatment. Numerous studies have shown that the HIF-1 signaling pathway is likely involved in promoting angiogenesis after ischemic stroke in the brain (Zhang et al., 2011). As a transcription factor in response to hypoxia, HIF-1 activity is increased in brains after ischemic attacks. In 1996, it was first reported that both subunit mRNAs of HIF-1 were upregulated in the brains of mice or rats when they were exposed to a hypoxic environment for 30 to 60 min (Jiang et al., 1996). Another study showed that HIF-1α expression was dramatically increased in the cerebral cortex of a rat after 60 min of recovery from cardiac arrest and remained boosted for more than 10 h. In addition, HIF-1α mRNA expression was fortified after focal ischemia in rat brain tissue. The increase was detected 8 h after the onset of ischemia and further elevated at 20 and 25 h (Zaman et al., 1999). These results demonstrate that the activity of HIF-1 is increased in ischemic brains and that the level of HIF-1α expression is heterogeneous. It has been reported that the HIF-1–mediated VEGF/Notch1 signaling pathway plays a crucial role in the development of angiogenesis in the ischemic brain. Apart from VEGF signaling, other complex mechanisms may also take part in HIF-1–mediated angiogenesis regulation after ischemic stroke. The expressions of angiogenesis–related genes such as the endothelin-1 (ET1), adrenomedullin (ADM), α1B-adrenergic receptor, nitric oxide synthase, Ang-2, stromal-derived growth factor-1 (SDF-1), PDGF-B, PlGF, and heme oxygenease-1 (HO-1) are also modulated by HIF-1 (Weih et al., 1999; Zhang et al., 2007; Yeh et al., 2008). In addition, HIF-1 mediated the regulation of collagen prolyl hydroxylase, MMPs, and plasminogen activator receptor and inhibitor (PAI) expression, which further modulates matrix metabolism and vascular maturation in the ischemic brain (Zou et al., 2018).

Because of the potentially pivotal roles in promoting angiogenesis by HIF-1 after ischemic stroke, it has been recommended that upregulation of HIF-1 activity is a highly promising therapeutic strategy for ischemic brain injury. Thus, the mechanism of HIF-1–induced angiogenesis in ischemic cerebral tissue has drawn much attention and is under extensive exploration. Currently, the only FDA-approved therapy for focal occlusive ischemia in the brain is the administration of the thrombolytic agent tissue plasminogen activator (tPA), which may have the risk of bleeding complications (Ohsawa et al., 2005). Thus, it is imperative to develop additional approaches to enhance therapeutic safety in ischemic stroke treatment. In recent years, several studies have raised great interest on the role of HIF-1 activation in the prognosis of ischemic stroke and whether upregulation of HIF-1 could benefit this disease. Therapeutic activation of HIF-1 applied before the ischemic stress or in the peri-ischemic period may theoretically enhance the natural response of angiogenesis in ischemic stroke patients. Some strategies have been used successfully on experimental activation of HIF-1 in ischemic disease animal models. For example, knocking out the central ODD domain will promote the activity of HIF-1α. The expression of such an HIF-1α transgenic protein in mouse models leads to significant activation of HIF-1 transcriptional targets and angiogenesis (Lin-Holderer et al., 2016). In addition, those neovessels are not leaky, and the intensive vascularity will not induce edema. This result contrasts with that of another study of VEGF therapy wherein edema is frequently detected and demonstrates that HIF-1 activation might keep away from this potential side effect in ischemic disease treatment (Ryou et al., 2015). Other studies have tried to use genetic therapy targeting to activate HIF-1 in the rabbit hind limb ischemia model and rat myocardial infarction model; these therapies improved angiogenesis and increased blood flow to the ischemic area (Li et al., 2016). Another focus on improving HIF-1–induced angiogenesis is suppressing the degradation of HIF-1α. For example, a macrophage-derived peptide called PR39 can interact with the proteasome and inhibit HIF degradation. Animal experiments have confirmed that PR39 treatment can improve peri-infarct angiogenesis in ischemic cardiac tissue (Hao et al., 2009). Besides using proteasome inhibitors, overexpression of peptides corresponding to the VHL-binding prolyl hydroxylation sites in HIF-1 also inhibits the degradation process of HIF-1α and further enhances angiogenesis in ischemic tissue. Another combined treatment with transgenic stem cells was applied in ischemic stroke rats. Rat bone marrow–derived mesenchymal stem cells were transfected with adenovirus containing HIF-1α genes with mutations at Asn 803 and Pro 564 sites, which prevent HIF-1 degradation. The cells with transgenic genes were injected into the cerebral artery occlusion of rats. After a week, improved angiogenesis and reduced infarction in brain tissue were observed; the rats’ ischemic stroke symptoms were also relieved (Li C. et al., 2017). HIF prolyl 4-hydroxylase domain proteins (PHD) are among the most pivotal inhibitors of the HIF-1 pathway. Suppression of the HIF-1 PHD by small molecular agents or genetic therapy may also inhibit HIF-1 degradation and activate the downstream gene’s transcriptional activity (Liu Y. et al., 2018). One study verified that PHD ablation in neurons improved ischemic stroke recovery in mice through endogenous adaptive angiogenesis by activation of the HIF-VEGF signaling (Mi et al., 2018). In general, the activation of HIF-1–dependent angiogenesis may provide therapeutic potential in ischemic and hypoxic cerebrovascular diseases. The central role of HIF-1 in the modulation of the hypoxia-correlated pathway has provided a promising approach for the development of novel therapeutic agents for ischemic stroke.

## Chinese Medicines Mediate Angiogenic Factors to Promote Angiogenesis by Regulating the Hif-1 Pathway After Ischemic Stroke

Herbal medicines, including herbal formulas, herbal extract, and chemical ingredients, have been widely used in the treatment of cardiovascular and cerebrovascular diseases for centuries because of reduced side effects (Fan et al., 2017). Previous studies indicated that herbal medicines are often used as an alternative therapy for prevention, treatment, and rehabilitation interventions of ischemic stroke (**Table 3**). As an important component of cerebral angiogenesis in patients with ischemic stroke, there has been great attention in developing activators targeting the HIF-1 pathway. HIF-1 activation can be induced by regulation of one of the following pathways: HIF-1 mRNA expression, HIF-1 protein expression, or signal transduction pathways. **Figure 3** summarizes the treatment of ischemic stroke by herbal medicines that regulate HIF-1α to promote angiogenesis through the different mechanisms.

**Table 3:** Herbal Medicines and Their Molecular Targets Regulated by the HIF-1 Pathway in Ischemic Stroke.

Herb Medicines	Molecular Targets	Reference
Activators of HIF-1 mRNA and/or protein expression		
Total flavonoid extracts	HIF-1α protein expression	(He et al., 2018)
MLC901 (herbal extracts)	HIF-1α protein expression	(Gandin et al., 2016)
*Nigella sativa* (NS)	HIF-1α mRNA expression	(Soleimannejad et al., 2017)
Flos Farfarae	HIF-1α protein expression	(Hwang et al., 2018)
Xue-Fu-Zhu-Yu decoction	HIF-1α protein expression	(Lee et al., 2011)
Bu Yang Huan Wu decoction	HIF-1α mRNA and protein expression	(Chen Z. et al., 2018)
Taohong Siwu decoction	HIF-1α protein expression	(Yen et al., 2014)
Xing-Nao-Jing	HIF-1α protein expression	(Chen Y. et al., 2018)
Naotai recipe	HIF-1α mRNA and protein expression	(Chen et al., 2014)
Berberine	HIF-1α protein expression	(Zhang et al., 2012)
trans-Resveratrol	HIF-1α mRNA and protein expression	(Agrawal et al., 2013)
Ginsenoside	HIF-1α protein expression	(Gao et al., 2018)
Xanthohumol	HIF-1α protein expression	(Yen et al., 2012)
Puerarin	HIF-1α protein expression	(Chang et al., 2009)
Galangin	HIF-1α mRNA expression	(Wu et al., 2015)
Activators of signal transduction pathways		
Catalpol and puerarin	PI3K-AKT-mTOR, ERK	(Liu et al., 2017)
Hinokitiol	PI3K-AKT	(Jayakumar et al., 2013)
Salidroside	PI3K-AKT	(Wei et al., 2017)
Silibinin	AKT-mTOR	(Wang et al., 2012)
Andrographolide	PI3K-AKT	(Chern et al., 2011)
Ginkgolide K (GK)	JAK2-STAT3	(Chen M. et al., 2018)
Shengui Sansheng San (SSS)	AKT-mTOR	(Liu B. et al., 2018)
Huang-Lian-Jie-Du-Tang	PI3K-AKT	(Zhang et al., 2014b)

### Activators of HIF-1 mRNA and/or Protein Expression

Several Chinese herbal medicines that target upregulating HIF-1 mRNA and/or protein expression have proangiogenic effects in ischemic stroke treatment. Ginsenoside, a major active ingredient of ginseng, has been demonstrated to be effective in the treatment of acute ischemic stroke. Gao et al. (2018) found that ginsenoside has therapeutic effects on cerebral ischemia and hypoxic injury through the HIF-1α–VEGF pathway in an oxygen-glucose deprivation/reperfusion (OGD/R) model of neural stem cells (NSCs). Xanthohumol, an ingredient of beer, is the principal prenylated flavonoid in hops (*Humulus lupulus* L). Yen et al. reported that xanthohumol-induced neuroprotection is associated with many factors such as HIF-1α, iNOS, and TNF-α. He et al. established a rat model of transient middle cerebral artery occlusion (tMCAO), followed by 24 h of reperfusion (Yen et al., 2012). Administration of total flavonoid extracts (TFC) could improve neurological deficits, reduce infarct volume, and promote angiogenesis by increasing the expression of HIF-1α, VEGF, Ang-1, Dll4, Notch1, and CD31 (He et al., 2018). MLC901, an herbal extract preparation modified from the TCM herbal formula, has been proven to have neuroprotective and neurorestorative properties in preclinical models of stroke, traumatic brain injury, and global cerebral ischemia. Gandin et al. (2016) found that 5-week pretreatment with MLC901 in MCAO-induced cerebral ischemia mouse models could regulate the expression of HIF-1α and its downstream targets, such as VEGF, Ang-1, and Ang-2. Chen et al. showed that administration of TCM formula Bu Yang Huan Wu (BYHW) decoction decreased cerebral edema, the neurological deficient score, and brain infarct volume in a rat model of cerebral ischemia/reperfusion (I/R) injury. Furthermore, BYHW treatment markedly decreased the mRNA and protein levels of HIF-1α and VEGF compared with those of the model treatment (Chen Z. et al., 2018). *Nigella sativa* (NS) is one of the widely used herbs from the family ranunculaceae. Soleimannejad et al. (2017) found that the *N. sativa* extract was associated with increased expression of VEGF and HIF-1α, markers of brain angiogenesis after total cerebral ischemia in rats. Puerarin, a major isoflavonoid isolated from the Chinese medicinal herb *Radix puerariae* (kudzu root), is widely used for treating cardiovascular disease in clinics. Chang et al. (2009) used a tMCAO rat model to study the effects of puerarin. Administration of puerarin inhibited the expression of HIF-1α, TNF-α, iNOS, caspase-3, and many factors, and it may be an ideal therapeutic measure after ischemia-reperfusion brain injury. Xue-Fu-Zhu-Yu decoction (XFZYD) is a traditional Chinese medicine formula widely used in cardiovascular diseases. Lee et al. found that XFZYD administration slightly reduced infarct volume compared with that of solvent-treated rats. However, the combination of XFZYD and recombinant tissue plasminogen activator (rt-PA) significantly reduced the infarct volume in cerebral ischemic areas. In addition, rt-PA administration significantly reduced the expression of TNF-α and iNOS but did not decrease the expression of HIF-1α or caspase-3, whereas XFZYD administration significantly reduced the expression of all these proteins in the ischemic region. In addition, XFZYD administration significantly enhanced the reduction of rt-pa–mediated TNF-α, iNOS, HIF-1α, and active caspase-3 expression (Lee et al., 2011). Galangin, a commonly used antioxidant, is a natural flavonoid derived from the rhizome of *Alpina officinarum* Hance. Wu et al. (2015) showed that galangin could promote angiogenesis and vascular remodeling to improve neurological function scores and the cerebral infarct area by upregulating the Wnt/β-catenin and HIF-1α/VEGF signaling pathway in a MCAO rat model. Herbal medicines targeting HIF-1 mRNA and/or protein expression are provided in **Table 2**.

### Activators of Signal Transduction Pathways

Several studies have shown that Chinese herbal medicines can also target different signal transduction pathways to upregulate HIF-1-induced angiogenesis. Huang-Lian-Jie-Du-Tang (HLJDT) is a classical heat-clearing and detoxicating formula of traditional Chinese medicine. Zhang et al. found that HLJDT preconditioning in the MCAO rat model could decrease the cerebral infarction volume, neurological deficient score, and cerebral water content. In addition, HLJDT preconditioning in cerebral cortical neurons *in vitro* under oxygen and glucose deprivation (OGD) could increase HIF-1α, VEGF, and erythropoietin (EPO) expression levels and activation of the PI3K/AKT signaling pathway (Zhang et al., 2014b). Shengui Sansheng San (SSS), a traditional Chinese herbal formula, has been used for stroke for more than 300 years. B. Liu et al. (2018) showed that SSS could activate AKT/mTOR/HIF-1α and ERK1/2 signals to facilitate VEGF production, resulting in angiogenesis after stroke in the rat MCAO model. Ginkgolide K (GK) is an extract isolated from the leaves of *Ginkgo biloba*. Chen et al. have used a tMCAO mouse model to verify the pharmacological properties of GK. GK treatment could significantly increase the expressions of HIF-1α and VEGF in the tMCAO model. In the OGD/R model of bEnd.3 cells, GK-induced upregulation of HIF-1α and VEGF could be eliminated by JAK2/STAT3 inhibitor AG490 (Chen et al., 2018). Andrographolide is a bicyclic diterpenoid lactone from the leaves of *Andrographis paniculata* (Acanthaceae). Chen et al. suggested that andrographolide could ameliorate brain injury in ischemic stroke by PI3K/AKT–dependent activation of the NF-κB and further activation of HIF-1α pathways *in vivo* and *in vitro* (Chern et al., 2011). As a conclusion, the herbal medicines targeting HIF-1 signal transduction pathways are provided in **Table 2**.

## Conclusions and Future Directions

HIF-1–induced angiogenesis has been involved in numerous pathological conditions, and it may be harmful or beneficial depending on the types of specific disease. Since the 1970s, the exploration on angiogenesis has sparked hopes in providing novel therapeutic approaches in multiple diseases with high mortality rates, such as cancers and ischemic stroke. Depending on different types of diseases and the expected treatment effects, angiogenesis-targeted therapies have different approaches. Generally, the clinical application of angiogenesis can be classified into two different strategies: antiangiogenesis (cancer) and proangiogenesis (ischemic stroke). The induction of angiogenesis for therapeutic purposes in ischemic stroke can be directly stimulated by various angiogenic factors, such as PlGF, VEGF, PDGF, and FGF, some of which have been applied in preclinical and clinical studies. However, treatments only using proangiogenic factors to induce angiogenesis were proven to be insufficient in ischemic disease; thus, novel treatments that can stabilize neovascularization with high-efficiency are required for better therapeutic effects. Therefore, HIF-1–induced angiogenesis may be a promising strategy for ischemic cerebrovascular disease. HIF-1 activation in ischemic cerebrovascular disease leads to a more mature and stable vascular formation compared with that of traditional proangiogenic factor therapy, wherein neovascularization tends to be leaky. Instead of proangiogenesis in ischemic stroke therapy, cancer treatments are based on suppression of angiogenesis for inhibiting tumor growth and metastasis. Current therapies are focused on suppressing VEGF activity, such as sunitinib (VEGFR2 inhibitor) and bevacizumab (VEGF inhibitor) target therapy. Because of the pivotal role of the HIF-1 pathway in modulating the activation of various proangiogenic factors in cancers, HIF-1 has been considered as a promising target for developing novel anticancer agents. Suppression of HIF-1–dependent angiogenesis involves the modulation of HIF-1 activity by regulating HIF-1α transcription and protein translation, HIF-1α DNA binding, HIF-1α and HIF-1β dimerization, and HIF-1 degradation. Considering the profound impact of HIF-1 on cancer progression and the unsatisfactory efficacy of current treatment protocols, several clinical trials are being conducted with potential antiangiogenesis agents that involve protein degradation, downregulation, or inactivation of HIF-1. It is noteworthy that, within a single herb concoction, sometimes we can find both inhibitors and activators of HIF-1, which will complicate the use of herbal medicines under clinical conditions. For example, ginsenoside Rg3 and ginsenoside Rg1 are both natural triterpenoid saponins extracted from red ginseng. Previous studies showed that ginsenoside Rg3 could inhibit tumor angiogenesis by decreasing the expression of HIF-1 in various cancers, whereas ginsenoside Rg1 might inhibit myocardial ischemia and reperfusion injury by activating HIF-1 (Chen et al., 2010; Yuan et al., 2019).

With a long history of more than 2,000 years of clinical use, Chinese herbal medicine is emerging as a complementary and alternative choice for its multitargeted, multileveled, and coordinated intervention effects against complex disorders, such as cancer and ischemic stroke. Research results from many *in vitro* and *in vivo* studies have demonstrated that several Chinese herbal formulations, herbs, or herbal compounds can induce or inhibit angiogenesis through multiple cellular mechanisms. Numerous preclinical studies have provided supportive evidence for using Chinese herbal medicines as a novel antiangiogenesis therapy for cancer or proangiogenesis therapy for ischemic stroke by targeting the HIF-1 pathway. However, the overall scientific evidence to back the application of Chinese herbal medicines for the management of cancer and ischemic stroke remains limited, and the results of these researches are sometimes contradictory and inconclusive. The underlying reasons for these inconsistencies include the complex chemical and pharmacological properties of Chinese herbal medicines and the interactions between the multiple bioactive ingredients of Chinese herbal medicines. More researches are needed to gain a better understanding of the dual effects of Chinese herbal medicines on angiogenesis in cancer and ischemic stroke treatment. In addition, despite the long history of Chinese herbal medicines in the treatment of cancer and ischemic stroke, well-controlled clinical studies with herbal medicinal products used for treating these diseases are still limited. More rigorously designed, controlled, randomized, international, multicenter clinical trials are urgently required for further validating Chinese herbal medicine efficacy in cancer and ischemic stroke treatment. Finally, as a double-edged sword, the important role of HIF-1 in angiogenesis should be considered as a promising target for treating cancer or ischemic stroke. The possible side effects and potential risk of angiogenesis-related complications by Chinese herbal medicines should also be considered when applying the HIF-1 target strategy for management of ischemic stroke and cancer.

## Author Contributions

MH and HS wrote the manuscript. NW, H-YT, QW, and YF revised the manuscript.

## Funding

This work was supported by the National Natural Science Foundation of China (No. 81673627), Guangzhou Science Technology and Innovation Commission Research Projects (201805010005), Research Grant Council, HKSAR (Project code: RGC GRF 17152116), and Commissioner for Innovation Technology, HKSAR (Project code: ITS/091/16FX).

## Conflict of Interest Statement

The authors declare that the research was conducted in the absence of any commercial or financial relationships that could be construed as a potential conflict of interest.
